# Diversity of Giant Viruses Infecting *Vermamoeba vermiformis*

**DOI:** 10.3389/fmicb.2022.808499

**Published:** 2022-04-22

**Authors:** Khalil Geballa-Koukoulas, Bernard La Scola, Guillaume Blanc, Julien Andreani

**Affiliations:** ^1^MEPHI, APHM, IRD 198, Aix Marseille University, IHU-Méditerranée Infection, Marseille, France; ^2^Aix Marseille Université, Université de Toulon, CNRS, IRD, MIO UM 110, Marseille, France

**Keywords:** *Vermamoeba vermiformis*, kaumoebavirus, faustovirus, tupanvirus, yasminevirus, orpheovirus, fadolivirus, clandestinovirus

## Abstract

The discovery of Acanthamoeba polyphaga mimivirus in 2003 using the free-living amoeba *Acanthamoeba polyphaga* caused a paradigm shift in the virology field. Twelve years later, using another amoeba as a host, i.e., *Vermamoeba vermiformis*, novel isolates of giant viruses have been discovered. This amoeba–virus relationship led scientists to study the evolution of giant viruses and explore the origins of eukaryotes. The purpose of this article is to review all the giant viruses that have been isolated from *Vermamoeba vermiformis*, compare their genomic features, and report the influence of these viruses on the cell cycle of their amoebal host. To date, viruses putatively belonging to eight different viral taxa have been described: 7 are lytic and 1 is non-lytic. The comparison of giant viruses infecting *Vermamoeba vermiformis* has suggested three homogenous groups according to their size, the replication time inside the host cell, and the number of encoding tRNAs. This approach is an attempt at determining the evolutionary origins and trajectories of the virus; therefore, more giant viruses infecting *Vermamoeba* must be discovered and studied to create a comprehensive knowledge on these intriguing biological entities.

## Introduction: The Rise of Amoebal Virology

Much of the history of virology has been devoted to the study of viruses infecting organisms of interest to humankind, including human viruses themselves (Mehle et al., 2018; Loeffelholz and Fenwick, 2019), but also crop and livestock viruses (Chen et al., 2021). However, the fact that emerging diseases may be triggered by viruses infecting wild hosts and the increasing societal awareness of the importance of environmental preservation has fueled the birth of environmental virology (Gollakner and Capua, 2020; Haider et al., 2020). This has broadened our understanding of the viral world, especially of viruses that infect microbes. It is now universally recognized that viruses are the most abundant biological entities on the Earth (Jian et al., 2021) and have contributed substantially to the evolution of life. In this general context, the scientific community began to take an interest in amoeba viruses in the early 2000s. This specific niche was only a marginal part of virology of interest to only a few specialists.

This discipline got off to a flying start with the accidental discovery of Acanthamoeba polyphaga mimivirus (La Scola et al., 2003) the first giant virus (GV) which dismantled some of the most historical dogmas in virology. For the first time, a virus was shown to have a particle size (450 nm) comparable to the size of a small bacterium and was observable using light microscopy, thus abolishing the old dogma that viruses are sub-microscopic in size (Claverie and Abergel, 2016). Following this discovery, over the next decade, a “race” between several research groups took place to isolate and study other giant viruses using *Acanthamoeba* strains as prey. As a result, *Acanthamoeba* spp. are now the protist organisms for which the most remarkable diversity of viruses have been characterized (Abergel et al., 2015; Brandes and Linial, 2019).

As of May 2021, 51 GenBank entries corresponded to either partially or fully sequenced genomes of viruses isolated from an *Acanthamoeba* host. All these viruses have a double-stranded DNA genome. Most *Acanthamoeba* viruses are recognized or possible members of the *Nucleocytoviricota* phylum [also known as the nucleocytoplasmic large DNA viruses (NCLDV)]. They are classified in at least seven groups of *Nucleocytoviricota* (including two official taxonomic families: *Mimiviriridae* and *Marseilleviridae*), and five genera are still awaiting official classification: “Pandoravirus” (Philippe et al., 2013), “Pithovirus” (Legendre et al., 2014), “Medusavirus” (Yoshikawa et al., 2019), “Pacmanvirus”(Andreani et al., 2017) Mollivirus (Legendre et al., 2015) and one genus of *Lavidaviridae* («Sputnikvirus»). Most *Nucleocytoviricota* members have genome sizes > 350 kbp and particle sizes > 250 nm, with pandoravirus salinus holding the current record for genome size among viruses of almost 2.5 Mb (Philippe et al., 2013) and pithovirus the record for particle size (1,500 nm) (Legendre et al., 2014). Although each giant virus encodes hundreds or thousands of distinct proteins, they share at the most only three universally conserved genes (Colson and Raoult, 2010). A recent phylogeny-centered study proposed that phylum *Nucleocytoviricota* may be portioned in six orders comprising 32 families embracing all large DNA viruses including giant viruses (Aylward et al., 2021).

At the other end of the scale, virophages (with a particle size of 50–75 nm) (*Lavidaviridae* family), which parasitize the viral factories of viruses in the *Mimiviridae* family replicating within *Acanthamoeba* hosts, have genome sizes of around 17–18 kbp and encode about 20 genes (Desnues et al., 2012; Gaia et al., 2014). In addition, yaravirus, isolated from *A. castellanii*, represents a divergent lineage of viruses with a 45 kbp genome that potentially lies outside the *Nucleocytoviricota* phylum (Boratto et al., 2020). The successful use of *Acanthamoeba* strains as bait for isolating original viruses has led some research groups to diversify the panel of potential prey in their isolation protocols, in order to continue exploring the diversity of protist viruses. Thus, a mimivirus-like giant virus, namely, Platanovirus saccamoebae, has been isolated from a natural strain of *Saccamoeba lacustris* (Amoebozoa, Tubulinea), itself previously isolated from the surface of a Platanus tree (Hauröder et al., 2018). The Microbe, Evolution, PHylogeny and Infection (MEPHI) group at the Institut Hospitalo-Universitaire (IHU)-Méditerannée Infection has tested alternative protist species, including the cosmopolitan amoeba *Vermamoeba vermiformis*, and has refined its virus isolation protocols to allow high throughput screening of environmental samples (Khalil et al., 2016). Like *Acanthamoeba* spp., *Vermamoeba vermiformis* has proven to be particularly effective in “fishing for viruses,” with 26 viral isolates characterized to date using this approach. This review aims to present the diversity and some of the peculiar characteristics of these viruses.

## *Vermamoeba Vermiformis* as Host Cell Support: A Novel Interest

The preference for using the Acanthamoeba protist in the viral co-culture strategy is historically due to the possibility of growing it under axenic conditions (PYG medium). Moreover, this strategy includes a step of antibiotic addition to eliminate bacterial contamination in the culture. Further, virus isolation attempts were successful after altering the medium substance in the relative *Acanthamoeba’* strains. One example was the substitution of gentamicin to amoxicillin in *A. castellanii*, which established another medium (PPYG/NaC) for growing amoeba (La Scola, 2014). This strategy was found to be highly prolific as *Acanthamoeba’s* strains successfully isolated many viruses. Other successful viral isolation approaches used their natural hosts, such as Cafeteria roenbergensis virus (Fischer and Hackl, 2016) and Bodo saltans virus (Deeg et al., 2018).

Since 2015, a new strategy for isolating viruses from amoeba co-culture was implemented using Vermamoeba, a protist that belongs to the same order as the genus *Acanthamoeba*, the *Amoebida* (Khan, 2006). This strategy included ameliorating the antibiotic and the antifungal composition, as *Acanthamoeba* strains are more biocides-resistant than *Vermamoeba* (Fouque et al., 2015b), eliminating the potential bacterial and fungal contaminants, replacing the Page’s amoeba saline solution used for *Acanthamoeba* with a novel one named “starvation medium adapted to *Vermamoeba vermiformis*,” and switching from daily microscopic observations to flow cytometry for faster processing and detection of the cytopathic effect in *Vermamoeba*.

This new approach opened the way for the isolation of new viral strains in an automatic way (Bou Khalil et al., 2016a). At the same time, the observation that *Vermamoeba* endoparasites can be located outside vacuoles after phagocytosis and that *Vermamoeba’s* rapid encystment process and its failure to survive in Page’s amoeba saline solution, resulted in *Vermamoeba* being used as an alternative amoeba host for viral capture (Bou Khalil et al., 2016b). It is worth mentioning that not all amoebas seem to be suitable hosts for the isolation of new viruses. This seems to be the case for the amoeba *Willaertia magna* (Boudjemaa et al., 2020).

The *V*. *vermiformis* was first described in 1967 by F.C. Page as *Hartmanella vermiformis* (Page, 1967). In 2011, this organism was reclassified and renamed under its current name due to the formation that differentiates it from other *Hartmanella* spp. (Smirnov et al., 2011). This dissimilarity is the cylindrical shape of *Vermamoeba* as well as the length/width ratio in comparison with *Hartmanella* (Park, 2016). The taxonomic change was later confirmed through 18S rRNA gene analysis of different protists (Delafont et al., 2018). *V. vermiformis* thrives in soil (Maisonneuve et al., 2016) but occurs more frequently in water (Dillon et al., 2014; Buse et al., 2017). In addition, *Vermamoeba* has been reported in snow, tap water (Javanmard et al., 2017), thermal waters (Reyes-Batlle et al., 2016), cooling towers, hospital, household sewages, industrial composts, and bioaerosols, in mammals, birds (Masangkay et al., 2018), and humans (Cabello-Vílchez et al., 2014; Masangkay et al., 2018). Regarding humans, *V*. *vermiformis* was reported to be associated with keratitis (Fouque et al., 2015a), an eye disease (Siddiqui et al., 2021), and indirectly to Legionnaires’ disease (Park, 2016), a disease that infects the human respiratory system (König et al., 2019). Likewise, *Vermamoeba* served as a dwelling for pathogenic bacteria (develop endosymbiotic attributes; Scheid, 2019) such as *Stenotrophomonas maltophilia* (Cateau et al., 2014), *Candidatus Rubidus massiliensis* (Pagnier et al., 2015; Bou Khalil et al., 2016b), *Bacillus anthracis*, *Pseudomonas aeruginosa*, *Legionella* spp., *Neochlamydia hartmannellae*, *Waddlia* as well as *Chlamydia*-*like* endosymbionts (Masangkay et al., 2018) and *Mycobacterium chelonae* (Cabello-Vílchez et al., 2014).

The life cycle of *V. vermiformis* comprises two stages (Baron, 1996) including the trophozoites stage and the cyst stage (Fouque et al., 2015a; Masangkay et al., 2018). The trophozoite consists of an elongated cylindrical motile form, which uses pseudopods to move (Fouque et al., 2015b), and allows them to feed and replicate (Dobrowsky et al., 2016). The *V. vermiformis* cell is mononuclear with a central dense and homogenous karyoplasm and several mitochondria with tubular cristae spread over cytoplasm (Fouque et al., 2015b). The cyst is spherical in shape, with a two-layer cell wall containing proteins, a small amount of glucose polymers, and an absence of cellulose and ostioles (Delafont et al., 2018). The encystment process lasts 9 h (Fouque et al., 2015a), and involves the locomotive form which is implicated in the cyst clustering (Delafont et al., 2018). Encystment occurs in a hostile environment such as one which is depleted of nutrients and under osmotic pressure (Fouque et al., 2015a). This situation leads to the formation of the cyst wall (between the sixth and the ninth hour of the encystment procedure) with a two-layer composition (Fouque et al., 2015b). This composition is responsible for the spherical form of *Vermamoeba vermiformis* (Fouque et al., 2015b) and the extreme conditions resilience (nutrient starvation, heat, cold, desiccation, and biocidal treatments) (Dobrowsky et al., 2016). Its form consists of monopodial long slug-like cells, with some granular materials in the cytoplasm with a slight anterior hyaline zone, and are influenced by temperature, pH, and osmotic pressure (Fouque et al., 2014). These cells can change to bi- or multipodial when they change their directions (Delafont et al., 2018). Studies showed that at 4°C, the formation is spherical and immobilized, while at 50°C, the trophozoite is lysed (Fouque et al., 2014). However, the cell lysis is slower than that of *Acanthamoeba* (Andreani et al., 2018).

To date, many *Vermamoeba* strains have been characterized from various parts of the world such as Iranian mineral springs (Feiz Haddad et al., 2020), Myanmar (Arab-Mazar et al., 2019), Spain (Canary Islands) (Reyes-Batlle et al., 2016), central and northern Italy, France, South Korea, the United States, Pakistan, England, and China (Montalbano Di Filippo et al., 2019). Only one draft genome sequence has been released, namely, the one from strain CDC-19. The reported genome sequence is 59,550,895 bp in cumulated length and encodes 22,483 genes with 41.7% G+C. Its analyses revealed the number of ORFans (i.e., putative ORFs without match in public sequence databases) estimated at 7,220, plus 2,829 genes with an unknown function. Phylogenomic analysis revealed that ten of them had the best homologous match in the Candidate Phyla Radiation bacterial group (Chelkha et al., 2020). This group includes more than 70 different bacteria phyla (Danczak et al., 2017). In addition, 185 genes had best matches in viruses of the *Nucleocytoviricota* phylum, with the most extensive relationships with klosneuviruses and Bodo saltans virus, 101 and 69 genes, respectively (Chelkha et al., 2020). Furthermore, 1,680 genes were putatively involved in signal transduction, 1,208 genes in post-translational modification processes, protein turnover, and chaperones, and 622 genes in intracellular trafficking, secretion, and vesicular transport. Finally, 154 genes were identified as related to defense mechanisms. Regarding the possible origin of the genes, 55.9% had best matches in eukaryotes (19.8% of those eukaryotes were amoebas), 10.2% in bacteria (including proteobacteria, which was most represented, followed by bacteroidetes and cyanobacteria); 0.6% in archaea and 0.8% in viruses. Finally, two genes matched with Ralstonia phage phiRSL1, and the last one matched with Synechococcus phage S-SKS1. Another observation about *V. vermiformis* genome is that this amoeba encodes about 3.5 introns per gene (Chelkha et al., 2020).

### Vermamoeba*-*Infecting Viruses From the Phylum Nucleocytoviricota

A total of 26 viruses infecting *Vermamoeba* (25 lytic viruses and one non-lytic virus) have been reported from multiple countries around the world, including France, Senegal, Algeria, Brazil, Saudi Arabia, and Lebanon. The most thoroughly studied virus is faustovirus, as 17 viral strains have thus far been described. The remaining viruses consist of a single strain or two strains. Reflecting on future perspectives, as more giant viruses will be subsequently described, the amount of information available will proportionally increase.

#### Faustoviruses

The development of a high-throughput characterization strategy in 2015 was fueled by the rapid isolation of eight faustovirus strains (Benamar et al., 2016), viruses that are close in phylogeny terms to the African swine fever virus (ASFV) (Reteno et al., 2015). The ASFV is responsible for the homonymous disease (Rolland et al., 2019), resulting in the death of domestic pigs (Galindo et al., 2015; Mazur-Panasiuk et al., 2020; Urbano and Ferreira, 2020). Transmission takes place through blood-sucking arthropods such as ticks (Guinat et al., 2016); although there is currently no evidence that mosquitoes may participate in ASFV transmission (Bonnet et al., 2020).

In the same context, it is believed that faustoviruses may possibly be transmitted by hematophagous arthropods (mainly biting midges), which suck blood from rodents, cattle, and humans. This suggestion is confirmed by FV traces isolated on sera from cattle and humans and various organs removed from rodents such as kidneys, tissue, and brains (Temmam et al., 2015). Notably, as most faustoviruses (FVs) have been isolated from wastewater samples, it could be assumed that their presence is an indirect indicator of fecal contamination (Bou Khalil et al., 2016a; Colson et al., 2017).

The FV-E12 isolate, one of the first to be described, was reported to have a 466,265 bp genome with a G + C% content of 36% and 451 predicted genes. In total, 164 of them (1/3 of all predicted proteins) were detected by nano-2D-LC-MS/MS to be present in its virion, and most of them were shared with proteins encoded by other viruses in the *Nucleocytoviricota* (Reteno et al., 2015). In contrast, ASFV members typically encode about 150 (Dixon et al., 2013; Jia et al., 2017; Shen et al., 2021) to 167 genes (Wang et al., 2021), which is only one-third of FVs. Furthermore, even though that FVs and ASFV are phylogenetically related, only 12% of FV genes appear to be homologous with ASFV (Aherfi et al., 2016). At the same time, FVs’ genome are larger than ASFV’s one (Koonin et al., 2020).

The sample containing FV-E12 was collected from a sewage environment in Marseille, France. After E12 stain, using the same capture technique in *V. vermiformis*, additional 16 FV strains have been sequenced and annotated so far. In general, the FV genomes are reported to be between 455,803 and 491,024 in length, encoding up to 506 genes (Geballa-Koukoulas et al., 2020). None of the FVs was found to contain tRNA genes. Finally, like most viruses in the *Nucleocytoviricota*, FVs contain a high number of ORFans.

An important structural characteristic concerns its virion. Indeed, the FV capsid has a double protein shell. The outer shell is made up of a double jelly roll protein, but the inner shell has a structure that is distinct from other “classic” capsid proteins. Such a structure is described as unique among DNA viruses (Klose et al., 2016). The Major Capsid Protein (MCP) gene spans over a 17,000 bp region and contains a surprisingly high number of type-I introns (Colson et al., 2017).

The capsid is about 2,400 Å (mature particles) in diameter and is fibril-free with an icosahedral shape (Klose et al., 2016). In addition, the reconstruction based on cryo-electron microscopy (Cryo-EM) observations revealed a double layer membrane between the external and the internal protein shells, allowing the MCP shells’ to touch the internal shell at several points. This formation consists of a double protein shell that seems to be in contrast with all other dsDNA viruses (Cherif Louazani et al., 2018), as some genomic materials are located between the internal and the external protein shells. Furthermore, it is believed that this agility found in the FVs structure is possibly created by either the presence of small lipids (mass spectrometry detection) or by an extra membrane that is not detectable by Cryo-EM and which encloses and protects the viral genome (Klose et al., 2016).

Using the transcriptomic sequencing results of the FV-E12 along with a splice-aware mapper and a double-round alignment strategy, 26 splice junctions were identified. These splice junctions are nested in the region that encodes the MCP gene (Cherif Louazani et al., 2018). In addition, this region is in the central part of the genome. Several group I introns that formed in this region were also recorded. In our previous study, introns were confirmed in the MCP gene of all FV isolates (varying in number between 13 and 18 per isolate), while some of them encoded several intron-homing endonucleases. Based on this observation, we hypothesized that there might be a link between the intron-generation mechanism (the endonucleases serve as a mediator before FV radiation in *V. vermiformis*) and MCP size. Genomic analysis revealed that all FV genomes contain high levels of gene colinearity, an amassing of indels, and local rearrangements in the MCP gene (close to the central region of the genome) along with their extremities which exhibit a faster evolutionary rate in that genomic region. Currently, known isolates belong to three different lineages among faustoviruses. The E9 and M/L clades consist of six isolates, and the D includes four isolates (Geballa-Koukoulas et al., 2020).

The *V. vermiformis* infection begins about 30 min after the first contact with the FV, while the *Vermamoeba’s* phagosomes can detect FV’s particles between 2 and 4 h after the infection. The eclipse phase is completed between the fourth and sixth hours after the beginning of infection, while the cell’s burst is ready to begin from 16 to 18 h post-infection (h.p.i.). At 20 h after the initial infection, the *Vermamoeba’s* cell comes to its lysis (Reteno et al., 2015). On the other hand, FV-mariensis, a strain isolated from Pampulha Lagoon Brazil (Borges et al., 2019), seems to postpone the cell lysis by 4 h. This occurs possibly due to soluble factor, that was not able to impede the *V. vermiformis’* encystment, post-infection, in the trophozoites stage, which remains conserved to cysts, and viral replication has not occurred. The same soluble factors were also described in *A. castellanii*, and consist of Mg^2+^ ions. In FV-mariensis, this soluble factor is enhanced by ethylenediaminetetraacetic acid (EDTA). It was also found that some intracellular bacteria can withstand and benefit from amoebal encystment suggesting FV-mariensis as a potential candidate antiviral model (Borges et al., 2019). The FVs genomes were initially hypothesized to have a circular conformation (Benamar et al., 2016). However, subsequent PCR assays and careful examination of the genome assemblies suggested instead that the typical FV genome is linear, like virtually in all other members of the *Nucleocytoviricota* (Geballa-Koukoulas et al., 2020).

#### Kaumoebaviruses

Kaumoebaviruses (KVs) is the second virus that has been isolated from *Vermamoeba*. The word “Kaumoeba” stands for **K**ing **A**bdulaziz **U**niversity a**MOEBA** because the first member was collected from sewage waters in Jeddah, Saudi Arabia (Bajrai et al., 2016). This genus is currently not recognized by the ICTV (Koonin and Yutin, 2019), but phylogenetic reconstruction clusters it amidst ASFVs and FVs (Cherif Louazani et al., 2018; Rolland et al., 2019), suggesting that it is a genuine member of the *Asfarviridae* family (Geballa-Koukoulas et al., 2021a; Karki et al., 2021). This phylogenetic position is reflected in common features between those viruses (Rolland et al., 2019). More specifically, FVs and KV share the same morphology: an icosahedral (Colson et al., 2017) capsid of approximately 200 nm in diameter (Bajrai et al., 2016; Borges et al., 2019), the replication cycle lengths, and a spliced major capsid protein gene (Rolland et al., 2019), which encode a protein of about 500 AA in size (Geballa-Koukoulas et al., 2021b). Another significant aspect found is that the upstream regions of the core genes of ASFV, FVs, and KVs have TATATA and TATTA motifs (Oliveira et al., 2018), which resemble *Asfarviridae* promoter (Rolland et al., 2019) and support the hypothesis for a similar gene expression mechanism (Oliveira et al., 2019a; Rolland et al., 2019). In 2021, a second member of the KV group, strain “LCC10,” was isolated from La Ciotat in France. Their comparison revealed that the LCC10 genome is 11,030 bp larger and encodes 45 genes more than the original strain named “Sc.” Similar to other viruses in the *Nucleocytoviricota*, both strains of KVs encode numerous genes that have no detectable matches in any database. For KV-LCC10, these account for approximately 76% of their total genes, while for KV-Sc strain, ORFans are calculated to 67%. Finally, the number of unique genes is 98 for KV-LCC10 and 85 genes for KV-Sc. The GC content in both isolates is about 43%. Neither KVs encode any tRNAs (Geballa-Koukoulas et al., 2021b), a feature that is also found in all FVs (Geballa-Koukoulas et al., 2020). The MCP gene, like that of FV, is spliced by several type I introns, with some of them encoding homing endonucleases, which strengthen the hypothesis of a common ancestor between faustoviruses and kaumoebaviruses. KVs have two introns that encode homing endonucleases, and their replication cycle is complete after 20 h (Bajrai et al., 2016).

The KV genomes exhibited a significant gene strand bias over the two-thirds of genome length, over-represented in the positive strand, a unique feature among all members of the extended *Asfarviridae*. The mechanism supporting this phenomenon remains unknown, but there is no apparent link with the host cell (Geballa-Koukoulas et al., 2021b).

#### Orpheovirus

Orpheovirus (OV) was isolated from a rat stool sample collected in La Ciotat, France, in the same location where FV-LC9 was discovered (Andreani et al., 2018). The virus has an ovoid-shaped particle ranging between 900 and 1,100 nm in length and measuring 500 nm in diameter (Souza et al., 2019). In addition, it comprises an ostiole-like apex to the ovoid shape with a diameter ranging from 70 to 80 nm obstructed in a thick membrane, involving in the DNA delivery to the host cell (Andreani et al., 2018). The particle structure shared some morphological properties with cedratviruses, pithoviruses, and pandoraviruses (Souza et al., 2019). In addition, both phylogenetic and pan-genomic analyses reveal that cedratvirus and pithoviruses have a close relationship with OV. This double-stranded DNA virus genome size of 1.4 Mb is immense compared with FVs and KVs. That said, the GC% content is lower (about 25%), encodes 1,512 genes, of which more than half are annotated as ORFans (Andreani et al., 2018). The OV genome is estimated to be circular (Andreani et al., 2018).

Furthermore, a common feature of OV with KVs and FVs is the apparent lack of tRNA-coding genes (Souza et al., 2019). However, its MCP gene is different from KVs and FVs as it does not contain introns (Andreani et al., 2018). The replication cycle of OV in *V. vermiformis* is completed within 24–38 h, which seems to be longer compared to FV and KV. Remarkably, the virus enters to *Vermamoeba* cell by the process of phagocytosis. The eclipsed phase is observed during the fourth hour after the infection. The viral factories are detected at 14–16 h.p.i. The new virions fill the *V. vermiformis* cytoplasm after 20 h.p.i., and the cell bursts between 24 and 38 h.p.i, marking the end of the replication cycle (Souza et al., 2019).

#### Tupanviruses

Tupanviruses (TVs) clade consists of two isolated members from the Brazilian soda lake and soil sediments (Silva et al., 2019). Indicatively, tupanvirus soda lake (TV-SL) was isolated in 2014 from a soda lake in the Pantanal region with extreme high salinity and pH, and tupanvirus deep ocean (TV-DO) was isolated in 2016 from Campos dos Goytacazes sediment sample collected 3,000 m deep under the ocean (Rodrigues et al., 2019). Tupan stands for “God of Thunder,” an important mythological figure for indigenous south American tribes (Abrahão et al., 2018). Both viruses possess a linear genome and likely belong in the *Mimiviridae* family (Rodrigues et al., 2019). These viruses can both replicate in *Acanthamoeba* and *Vermamoeba* (Silva et al., 2019), and in other amoebas such as *Dictyostelium discoideum* and *Willaertia magna* (Rodrigues et al., 2019).

Tupanvirus strains were morphologically characterized and revealed an unusual structure (Silva et al., 2019), marked by the presence of a semi-icosahedral mimivirus-like capsid attached to an elongated cylindrical tail (de Miranda Boratto et al., 2019) of 550 nm in length (Rodrigues et al., 2019). The cylindrical tail may vary in size, so the overall length of some particles can exceed 2 μm. A mimivirus-like capsid has the stargate portal on one side, surrounded by fibrils (Silva et al., 2019). Even if the MCP structure is similar to the *Mimiviridae* members, their genome structure, ranking them as one of the largest (Oliveira et al., 2019a), distinguishes TVs from other mimivirids (Silva et al., 2019). With regards to their general features, TV genomes are much bigger in size compared with the rest of *Vermamoeba* viral isolates. Specifically, TV-SL genome is 1,439,508 bp, while TV-DO is 1,516,267 bp in size, and they encode 1,276 and 1,359 genes, respectively. In addition, TVs encode many tRNAs, with TV-SL encoding 67 and TV-DO three more. Tupanviruses encode a set of 20 aminoacyl-tRNA synthetases (aaRS) specific genes too. Their genomes also contain 11 translation-associated factors that are linked with tRNA/mRNA maturation, a ribosome modification protein, and a mannose-binding protein (Oliveira et al., 2019b). Curiously, TV-SL lacks the RNAse T2 gene, which plays an essential role in the RNA catabolism inside the cell (Rolland et al., 2020). Like many giant viruses, 26% of TV-DO genes (375 out of 1,276) and 28% of TV-SL ones (378 out of 1,359 genes) are annotated as ORFans (Abrahão et al., 2018). Until 2019 and the discovery of yasminevirus, TVs were the largest viruses infecting the *Vermamoeba* group (see section “Yasminevirus”).

A comparative genomic analysis between the TVs shows that, with exception of the terminal regions, in which the TV-SL shows local rearrangements and translocation compared to the TV-DO genome, the rest of their genomes are extremely conserved (Rodrigues et al., 2019). The cell replication starts almost instantly after the “adhesion” of the virus to the cell. This adhesion is believed to occur due to the mannose receptor gene expression. Also, it was reported that the expression of a mannose receptor gene participates in the pathogenesis of *Acanthamoeba* (Oliveira et al., 2019b). Tupanvirus replication time varies and depends on the eclipse phase time. For instance, for a 4-h post-infection eclipse phase, the replication cycle ends after 36 h.p.i., explaining why the final stage varies between 36 and 72 h (Silva et al., 2019).

#### Clandestinovirus

Clandestinovirus (CLV), the first non-lytic isolate on *V. vermiformis*, was recently described (Rolland et al., 2021). This virus was separated by a single cell microaspiration strategy (Sahmi-Bounsiar et al., 2019) from a mixed co-culture associated with another giant virus (clandestinovirus ST1 and FV-ST1) collected from an environmental sample. Clandestinovirus has an icosahedral capsid devoid of fibrils and has a virion size between 175 and 202 nm in diameter. It possesses a linear genome of 581,987 bp (43.5% G + C content) assembled in two scaffolds which encodes 617 open reading frames, corresponding to a coding density of 86% (Rolland et al., 2021). In addition, it has one serine- and one histidine-like tRNAs. The clandestinovirus histidine-like (His-like) tRNA is also found in medusavirus. Indeed, according to the results of several analyses, these two viruses are phylogenetically related (Zhang et al., 2021).

The high-throughput automated technique detected the clandestinovirus in the *V. vermifomis* cytoplasm in which the fluorescence signal augmented after 24 h post-infection and remained up to 5 days confirming the non-lytic nature of viral infection (Francis et al., 2019). Specifically, after the viral entry into the *Vermamoeba* cell, the viral particles transposition from the cytoplasm to the nucleus to begin replication. This step was also observed during the replication of *Herpersviridae* (Jean Beltran and Cristea, 2014). Viral factories are observed 7–12 h.p.i, and at 16 h.p.i. The newly formed virions are discharged by exocytosis, which completes the replication cycle (Rolland et al., 2021).

#### Klosneuvirus-Like Viruses

The “klosneuvirus clade” consists of two viral isolates that infect *V. vermiformis*. yasminevirus and the fadolivirus, which is the newest member described. The main feature of these genomes is their large size, indeed the biggest among all the known *Vermamoeba*-infecting viruses.

##### Yasminevirus

One of the most recent GV isolates from *V. vermiformis* is the yasminevirus (YV). Like KV, YV was isolated from sewage water in Jeddah, Saudi Arabia (Bajrai et al., 2016). The structure and morphology of the mature YV particles are similar to the Bodo saltans virus, and its virion core is enclosed by putative thin membranes, which are surrounded by an icosahedral capsid of 330 nm in diameter. This icosahedral capsid structure is also found in most members of the phylum *Nucleocytoviricota* infecting *Vermamoeba*, such as KVs and FVs.

The capsid is surrounded by a layer of thin fibrils that mediate the start of the infection. Its mosaic genome structure is the biggest among all *Vermamoeba*-infecting viruses from the phylum *Nucleocytoviricota*, extending to 2,126,343 bp. The number of genes is predicted to be 1,541, which is the highest among *Vermamoeba*-infecting viruses belonging to *Nucleocytoviricota*. Comparative genomic analyses demonstrated that 34% of the genes have the best matches with those encoded by other viruses, 13.2% with those in Eukaryota, 7.2% in bacteria, and less than 1% in Archaea. The ORFans comprise about 44% of the total number of genes. Despite the YV genome being bigger than the TVs’, it encodes similar number of tRNAs (70 tRNAs in total) and contains 20 aminoacyl-tRNA synthetases (aaRSs) along with several translation and elongation factors. The GC content of YV was calculated to be 40.2%.

The replication cycle begins 30 min after infection initiation, particles can be observed in the host cytoplasm and, after a further 30–90 min, the cytoplasm is prepared for the eclipse phase during which the virus particles have vanished. This stage was further distinguished by the modification of the host cell nucleus and the development of intranuclear cleared regions. After 12 h.p.i., viral factories can be detected in the host cell, and the final cell lysis is completed after 24 h.p.i. (Bajrai et al., 2019).

##### Fadolivirus

Fadolivirus (FL) is the latest *Vermamoeba*-infecting isolate, described in 2020 from sewage water in Sidi bel Abbes in Algeria (Boudjemaa et al., 2020). This is a lytic virus with an icosahedral structure. The viral particles measured about 300 nm from which short fibrils extended on its capsid. Phylogenetically, this virus is clustered in a clade along with klosneuvirus, barrevirus, and indivirus.

Alike clandestinovirus, this virus has a linear genome and was assembled in two scaffolds totaling 1,595 Mbp (Andreani et al., 2021). It encodes 1,452 genes of which 38% have a detectable homology with other amoebal viruses, as well as 66 tRNAs and 23 aaRSs, a number that correlates with the yasminevirus that encodes 20. Moreover, 20 genes are shared with several related viruses of the “klosneuvirus clade” (core genes). The replication cycle of FL in *V. vermiformis* starts with phagocytosis. Up to 12 h.p.i, the eclipse phase is fully recognized. In total, 4–8 h after the eclipse phase, the viral particles started in the host cytoplasm, resulting in its final lysis after 36 h.p.i. (Andreani et al., 2021).

## Comparison of the *Vermamoeba*-Infecting Viruses From the Phylum *Nucleocytoviricota*

As many giant viruses have been described, different replication stages are grouped into three phases for a precise description and comparison. The first phase comprises the early stage of infection after phagocytosis. The second one includes the eclipse phase and the stage when the viral factories become distinguished in the host cell, and finally, the last phase comprises the advanced stage and the final stage, in which an entire replication cycle is completed.

Sorting genomes in order of genomic size, the largest one is YV with 2,126,343 bp, followed by FL, TV-DO, OV, TV-SL, and FVs with the shortest genomes in KVs. In the same line, YV seems to encode more genes while KVs encode the least number. Regarding the GC content, orpheovirus has the smallest percentage, and the KVs have the highest percentage. About the structure of the viral isolates, all viruses exhibit an icosahedral capsid except orpheovirus with an ovoid shape and tupanviruses with a more elaborate shape comprising a ∼550 nm long cylindrical tail covered with fibrils. Regarding the infection time in *V. vermiformis*, all viruses start to visibly affect *Vermamoeba* at around 4 h, except FL which starts after 12 h; however, FL is a non-lytic virus and thus has a different infection behavior. The complete replication cycle appears to be the slowest in TVs, while in KVs and FVs, it replicates the most quickly among all the viruses.

For the tRNAs, CLV, YV, TVs, and the non-lytic virus of FL are the only viruses that contain tRNAs. One important observation is that these viruses could have common ancestry, as they are significantly related on the phylogenetic level. Of course, additional elements are required to validate this hypothesis. The origin of tRNAs is highly connected with the replication time (Maizels and Weiner, 1994). Furthermore, it is reported that the tRNA gene is associated with the DNA replication time (Müller and Nieduszynski, 2017) as they impede the replication process at the S phase (Molla-Herman et al., 2015). Unfortunately, based on the current bibliography, the role of viral tRNAs in cell life cannot be determined.

**FIGURE 1:**
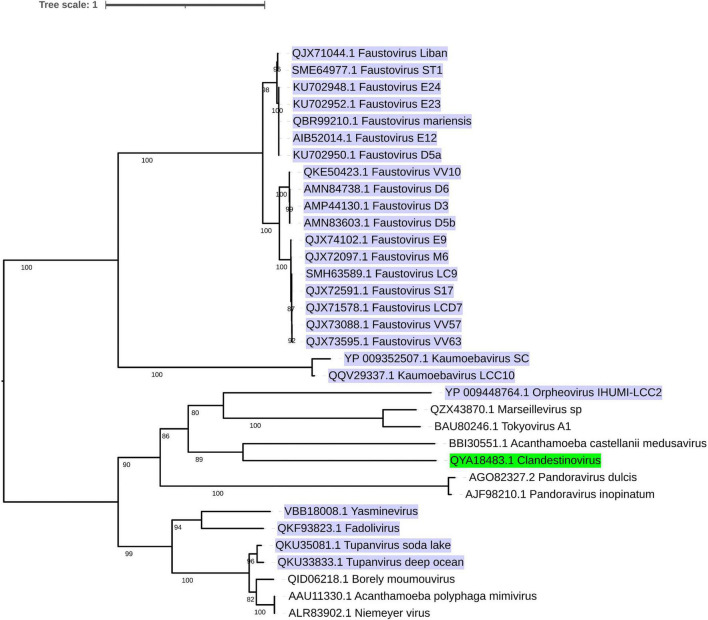
Phylogenetic tree of currently recognized and additional putative members of the phylum *Nucleocytoviricota* infecting *Vermamoeba* sp. based on DNA polymerase protein sequences. Amino acid sequences were aligned with MAFFT (Katoh and Standley, 2013) (with AUTO as parameter). The most conserved regions of the aligned sequences were selected by TrimAl (Capella-Gutierrez et al., 2009; v1.4.rev22 build (2015-05-21) with strictplus as a parameter]. IQ-TREE software (Nguyen et al., 2015; version 1.6.12 using -bb 1,000 and -m TEST as options) was applied for the tree reconstruction. Finally, ITOL (Letunic and Bork, 2021; version 10.0.5) was used for the visualization of the tree. Lytic viruses are highlighted in purple, while the non-lytic virus is marked in green.

Considering the aforementioned information and the phylogenetic relationship (Geballa-Koukoulas et al., 2021a; Figure 1) between those viruses, we propose that viruses that infect *V. vermiformis* could be categorized into three groups. FVs and KV could comprise the first, OV the second, and the remaining viruses could form the latest group. Unfortunately, to make our estimations more accurate, new data or, even better, new isolates need to be investigated. Finally, all *Nucleocytoviricota* infecting *Vermamoeba* isolates contain a considerable number of ORFans proteins. Table 1 summarizes the main features for *Vermamoeba*-infecting giant viruses (such as the genome length size, the G + C content, the number of predicted genes, the number of tRNAs along with the three phases of the replication cycle described earlier).

**TABLE 1:** General features of all *Vermamoeba*-infecting viruses, recognized and/or potential members of the phylum *Nucleocytoviricota*.

	Faustovirus*	Kaumoebavirus*	Orpheovirus	Tupanvirus*	Yasminevirus	Fadolivirus	Clandestinovirus
Genome length (bp)	455,803–491,024	362,586–350,731	1,473,573	1,439,508–1,516,267	2,126,343	1,573,504	581,987
G-C% contest	36.20–39.90%	43.1–43.7%	24.98%	28%	40.2%	27.10%	43.8%
Predicted Genes	471–506	429–507	1,199	1,276–1,359	1,541	1,452	617
First phase time	4–6 h	4 h	4 h	4 h	4 h	12–16 h	4–7 h
Second phase time	8–10 h	6 h	14–16 h	7–12 h	12 h	20–24 h	7–12 h
Final phase time	18–20 h	20 h	24–38 h	36–72 h	24 h	36 h	16 h
Number of tRNAs	0	0	0	68–70	70	66	2
Virion structure	Icosahedral	Icosahedral	Ovoid	Icosahedral with a tail	Icosahedral	Icosahedral	Icosahedral without fibrils
Lytic/non-lytic	Lytic	Lytic	Lytic	Lytic	Lytic	Lytic	Non-lytic
	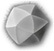	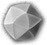	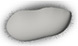	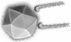	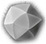	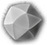	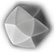
References	Geballa-Koukoulas et al., 2020	Bajrai et al., 2016; Geballa-Koukoulas et al., 2021b	Andreani et al., 2018	de Miranda Boratto et al., 2019	Bajrai et al., 2019	Andreani et al., 2021	Rolland et al., 2021

*Some general features, along with the replication time in the host cell. Three phases (first, second, and final) are “modified” to fill in the missing reference information. The putative genera that contain more than on isolate are mention with an asterisk.*

## Conclusion

Giant viruses (GVs) are a relatively new and up-and-coming niche of virology linked with the entire world of eukaryotes. Scientists, using the free-living amoebas succeeded in isolating GVs. This technique was initially adopted in 2003 for the isolation of a first GV from *Acanthamoeba polyphaga*. By applying the same process, many new isolates have been characterized ever since. *Acanthamoeba* spp. were mainly used to capture GVs until the faustovirus (E12 strain) isolation in 2015, which pioneered *V. vermiformis* as a model organism, which blazed a trail for new GVs discoveries.

Currently, a total of 26 giant virus isolates are known to infect *V. vermiformis*. Here, for the first time, we present a systematic comparison of this group of viruses. A total of nineteen viral isolates likely belong to the extended *Asfarviridae* family (17 FVs and 2 KVs), two belong to “klosneuvirus clade” (YV and FL), two are comprised in the *Mimiviridae* family (2 TVs), and other isolates to be yet officially classified of OV and the non-lytic virus of CL. Regarding the replication cycle of those viruses in *Vermamoeba*, FVs and KVs replicate at a faster rate compared to the rest of *Vermamoeba*-infecting viruses from the phylum *Nucleocytoviricota.*

In summary, the 26 *Vermamoeba*-infecting viruses belonging to the phylum *Nucleocytoviricota* are here reviewed and informally classified into three groups based on their size, replication time, and the number of tRNAs. The first group consists of KV and FVs. The second is represented by OV, and the last group contains TVs and YV. We understand that this grouping may not stand the test of time as it is based upon limited data available at present time. We intend to provide an initial point for further discussions as the number of characterized isolates of GVs increases over time.

The world of viruses from the phylum *Nucleocytoviricota* infecting *Vermamoeba* remains shrouded in mystery, and the comparison with similar viruses from other aquatic microorganisms such as algae is a challenge. The discovery of endogenous giant virus-derived sequences from several green algae has also been reported recently (Moniruzzaman et al., 2020), which opens new possibilities in studying and understanding these viruses. However, using *Vermamoeba* spp., seven viral isolates have been described, and using *Acanthamoeba* spp., the number of isolates was much higher. Moreover, the amoeba co-cultivation technique allows isolation and studying different viruses in mixed infections and their separation as described by the single-cell microaspiration strategy (clandestinovirus ST1 and FV-ST1). To conclude, the amoeba-isolation technique is a relatively simple, reliable, and efficient method for discovering novel giant viruses. The use of new protists as potential host models could also facilitate novel discoveries.

## Author Contributions

KG-K designed the figure and table, wrote the original draft, and edited the final version. BL, GB, and JA supervised this work and reviewed the manuscript. All authors read and approved this manuscript.

## Conflict of Interest

The authors declare that the research was conducted in the absence of any commercial or financial relationships that could be construed as a potential conflict of interest.

## Publisher’s Note

All claims expressed in this article are solely those of the authors and do not necessarily represent those of their affiliated organizations, or those of the publisher, the editors and the reviewers. Any product that may be evaluated in this article, or claim that may be made by its manufacturer, is not guaranteed or endorsed by the publisher.
